# Awake patients' experiences and coping needs during awake prone positioning in general wards: a patient-perspective qualitative study

**DOI:** 10.3389/fmed.2026.1768146

**Published:** 2026-04-24

**Authors:** Deyou Chen, Yongmei Chen, You Yuan, Yongjing Wen, Xiaopeng Wang, Xiaojuan Li, Qiong Wu

**Affiliations:** 1 Department of Radiology, Affiliated Hospital of Zunyi Medical University, Zunyi, Guizhou, China; 2 Department of Critical Care Medicine, Affiliated Hospital of Zunyi Medical University, Zunyi, Guizhou, China; 3 Cardiology Department, Department of Critical Care Medicine, Affiliated Hospital of Zunyi Medical University, Zunyi, Guizhou, China; 4 Respiratory Department, Department of Critical Care Medicine, Affiliated Hospital of Zunyi Medical University, Zunyi, Guizhou, China; 5 Department of Nursing, Affiliated Hospital of Zunyi Medical University, Zunyi, Guizhou, China

**Keywords:** awake prone position, demand, experience, nursing, qualitative research

## Abstract

**Objective:**

To investigate the experience and needs of awake prone positioning in patients, and to provide evidence to inform interventions aimed at enhancing patient comfort and optimizing awake prone positioning therapy.

**Methods:**

A qualitative descriptive research method was used to conduct face-to-face, semi-structured, in-depth interviews with 21 patients who underwent awake prone positioning at a tertiary hospital in Guizhou Province from January to December 2025. The Colaizzi 7-step analysis method was used to encode, summarize, and analyze the interview data.

**Results:**

Four major themes were extracted: (1) coexistence of physical comfort and discomfort; (2) differential psychological experience; (3) factors hindering prone position therapy; and (4) coping strategies and support needs.

**Conclusion:**

Patients undergoing awake prone positioning experience various physiological and psychological responses, including a lack of knowledge, inconvenience caused by iatrogenic tubing, and inadequate equipment. Patients are eager to receive thoughtful care, and medical staff need to attend to patients' feelings and needs, provide personalized interventions, improve comfort, and promote disease recovery.

## 1 Introduction

Awake prone positioning has become a critical therapeutic approach for patients with hypoxic respiratory failure (1–3). It has been shown to significantly improve blood oxygen saturation, enhance the oxygenation index, and effectively reduce the need for intubation (4, 5). This treatment method is simple, highly feasible, and associated with fewer complications, making it particularly suitable for patients who can perform it independently, without requiring extensive workforce or material resources (6, 7). As a result, awake prone positioning has been increasingly applied in the treatment of severe pneumonia, acute respiratory failure, and other conditions (8–11). Positioning strategies can improve both oxygenation and ventilatory mechanics. Cuenca-Zaldívar et al. demonstrated that using an orthostatic board in ventilated ICU patients increased tidal volume and GCS scores without adverse events (12).

Patient experience is a key indicator of healthcare quality. Focusing on patients' treatment experiences and needs not only improves the quality of nursing care but also significantly enhances patient satisfaction (13). While existing research primarily focuses on the impact of awake prone positioning on oxygenation and clinical outcomes, there is a lack of studies on patients' experiences and needs during treatment (14, 15). Therefore, this study, using semi-structured interviews, aims to analyze patients' experiences and needs during awake prone positioning in depth, providing evidence for healthcare professionals to develop personalized interventions that enhance patient comfort and promote recovery.

This study was conducted in the Zunyi area of Guizhou Province, in western China. Compared to other regions, the area is economically and medically underdeveloped, and resource limitations may affect patients' understanding and acceptance of treatment methods. When faced with new treatment approaches, patients may have doubts about their effectiveness and necessity. Through interviews, the study explores patients' treatment experiences within the constraints of their cultural backgrounds and limited resources. The findings provide real-world evidence and guidance to improve the quality of healthcare services and optimize treatment outcomes in the region.

## 2 Participants and methods

### 2.1 Study participants

Convenience sampling was used to select patients in the general wards of a tertiary-grade a hospital in Guizhou Province who underwent awake prone positioning from January to December 2025. Participants were screened by two research team members (Y. W. and X. W.). To ensure a diverse sample, we aimed to recruit patients with variation in age, sex, educational level, and occupation. Inclusion criteria: (1) age ≥18 years; (2) normal cognitive and verbal communication abilities; (3) awake, prone positioning duration ≥2 h per day; (4) provided signed informed consent. Exclusion criteria: (1) patients with unstable underlying medical conditions that could be exacerbated by the interview process or for whom the interview would interfere with ongoing treatment; (2) patients with mental disorders or those unable to cooperate; (3) patients with severe hearing or speech impairments that prevented effective communication; (4) patients who did not complete the full interview content, withdrew during the interview, or had an interview duration of less than 10 min; (5) patients who explicitly declined participation.

### 2.2 Research methods

This study employed a qualitative descriptive design. This approach is particularly suitable for directly exploring and describing participants' experiences, perceptions, and needs in a way that stays close to the data, without applying existing theoretical or philosophical frameworks.

#### 2.2.1 Interview outline development

The research team developed the interview outline based on the research objectives by reviewing relevant literature and discussing it with project team members. The interview outline is as follows: (1) how did you feel physically during the prone position therapy? Can you compare any changes in your condition before and after the prone positioning? (2) How did you feel emotionally during the prone position therapy? (3) Based on your treatment process, what factors affected your ability to undergo awake prone positioning? (4) If you experienced discomfort during the prone position, how did you handle it? What assistance did you require from medical staff and family members? (5) Is there anything else you would like to add about awake prone positioning?

#### 2.2.2 Data collection

A semi-structured interview method was used to collect data. The researchers had received systematic training in qualitative research methods and mastered the techniques of semi-structured interviews, which enabled them to conduct the interviews independently. (1) Before the interview, the researcher contacted the participant, introduced themselves, established a rapport, and explained the purpose, methods, and significance of the study. The interview time and location were scheduled, and informed consent was obtained. (2) During the interview, face-to-face interviews were conducted either in a quiet, private meeting room or at the patient's bedside during non-treatment periods, following confidentiality principles. All interviews were conducted in Chinese. The entire interview was recorded, and the researcher listened attentively, observed carefully, and adjusted the order and phrasing of questions based on the participant's responses. Non-verbal information, such as facial expressions and gestures, was also recorded. Ambiguous statements were rephrased, and follow-up questions were asked when needed. (3) Toward the end of the interview, the researcher asked, “Is there anything else you would like to share with me?” to gather additional information. Each interview lasted between 10 and 40 min. Data collection and analysis were conducted iteratively. After each set of three to four interviews, the research team discussed preliminary findings. By the 18th interview, core themes were well-developed with no new codes emerging across subgroups. Referring to sample sizes in similar studies (16–18), three additional interviews (P19-P21) were conducted to confirm saturation. No substantially new information emerged, indicating that meaning saturation had been achieved.

### 2.3 Data analysis

Data analysis was conducted using a thematic approach guided by Colaizzi's steps. This involved: (1) within 24 h after the interview, two researchers independently listened to the recordings, noted tone and pauses, and transcribed them into original drafts. These drafts were cross-checked for discrepancies and merged, resulting in a cleaned draft free of grammatical errors and missing words, anonymized, and marked with non-verbal information (2). The two researchers then jointly read the cleaned draft, identified all original segments directly related to awake prone position experiences, and assigned preliminary labels to these segments (3). Similar or recurring labels were clustered and refined into meaning units that were adequately supported by the original segments (4). The meaning units were further abstracted and optimized into concise phrases, retaining the original meaning, and mapped to preliminary themes such as physiological sensations, psychological experiences, barriers, and support needs (5). For each theme, detailed descriptions were written, incorporating key quotations to present the participants' perspectives clearly (6). If any ambiguity arose in the coding of the original statements or if there was disagreement in theme classification, a third researcher would facilitate group discussions. If necessary, qualitative research experts involved in implementing prone positioning would be consulted to refine the themes into a hierarchical framework with main categories and subcategories (7). Finally, the refined framework and representative quotations were returned to the participants for confirmation to ensure the results faithfully reflected their actual experiences before finalizing the analysis.

### 2.4 Researcher characteristics and reflexivity

The interviews were conducted by the first author (D.C.) and the second author (Y.C.). D.C. is a male respiratory rehabilitation therapist with 8 years of clinical experience in guiding patients through prone positioning. Y.C. is a female nurse with 12 years of extensive experience in respiratory care and holds a master's degree. Neither interviewer was involved in the direct clinical care of participants during hospitalization, which helped minimize power imbalances.

Before the study started, both interviewers had positive views on prone positioning based on their clinical experience. To reduce this potential bias, they kept reflexive journals throughout data collection and analysis, documenting their assumptions and discussing them with the research team. The corresponding author (Q.W.), who designed a prone positioning device for patients and holds a Chinese patent for its clinical use, took part in team discussions. A third researcher (Y.Y.), an ICU nurse with 8 years of experience managing many COVID-19 patients who underwent prone positioning and holding a master's degree, was not involved in data collection. He helped lead debriefing sessions to challenge emerging interpretations and offer an independent perspective.

### 2.5 Quality control

The study strictly adhered to qualitative research methods, and specific quality control measures included: (1) a maximum variation strategy was used to select representative study participants, covering different occupations, educational backgrounds, and ages. Interviews were conducted by the first and second authors of the manuscript, either jointly or separately, after participants completed prone positioning and reported no serious physical or psychological discomfort (2). Prior to the formal interviews, three patients were selected for pilot interviews. Based on their feedback, the interview outline was adjusted in sequence and wording to ensure it was easy for participants to understand (3). During the interviews, a relaxed atmosphere was maintained. Family members were allowed to accompany participants only if the participant wished. When present, they were encouraged to allow the participant to speak first and primarily, with the interviewer directing questions to the patient. In cases where family members contributed to the conversation, these contributions were noted in the transcripts but were not included in the primary analysis, which focused exclusively on the patient's own words and experiences (4). Within 24 h of the interview, a verbatim transcription was completed. The cleaned draft was sent back to the participants for verification. After cross-checking the text between two researchers, coding, inductive analysis, and theme refinement were conducted.

## 3 Results

In addition to the three patients in the pilot interviews, 28 eligible patients were approached during the study period; of these, 21 completed the interview and were included in the final sample (labeled P1–P21). Reasons for non-participation were fatigue or illness (*n* = 2), hearing impairment (*n* = 1), interview duration < 10 min (*n* = 2), withdrawal after initial consent (*n* = 1), and incomplete responses to the interview guide (*n* = 1). Among the 21 participants, 11 were male and 10 were female. The average age was 63.7 ± 12.4 years (range, 38–88 years). The participants' educational backgrounds were as follows: 7 had elementary school education or below (33.33%), 6 had junior high school education (28.57%), 4 had high school/technical secondary school education (19.05%), and 4 had college education or higher (19.05%). A total of 13 participants (61.90%) had education at or below the junior high school level. In terms of occupation, the majority were farmers/unemployed/migrant workers (11 participants, 52.38%), followed by workers/staff/sales (5 participants, 23.81%), and retirees/teachers/cadres (5 participants, 23.81%). The overall educational level of the sample was low, which is consistent with the characteristics of the population in western China. The general demographic information of the participants is shown in Table 1.

**Table 1:** General demographic characteristics of the participants.

No.	Gender	Age	Education	Occupation	Disease diagnosis	Sessions/ day	Session/ min	Therapy days
P1	Female	75	Technical secondary	Retired worker	Pulmonary infection	3–4	30–120	2
P2	Female	54	High school	Farmer	Severe pneumonia	2	240	10
P3	Male	42	Junior high	Sports coach	Pulmonary infection	2–6	120	6
P4	Male	54	College	Worker	Community-acquired pneumonia	1–2	60–120	5
P5	Male	86	Junior high	Administrator	Community-acquired pneumonia	4–5	30	5
P6	Male	72	Elementary	Farmer	Community-acquired pneumonia	3	30–60	3
P7	Male	69	Bachelor's	Teacher	COPD	2–3	60–120	2
P8	Female	65	High school	Civil servant	Pulmonary infection	3	60	2
P9	Male	88	Technical secondary	Retired official	Respiratory failure	4–5	30	3
P10	Female	53	Below elementary	Farmer	Severe Pneumonia	3	60–120	2
P11	Male	84	Elementary	Farmer	Community-acquired pneumonia	4–5	30–60	4
P12	Female	45	Junior high	Staff	Respiratory failure	3	60–120	5
P13	Male	79	College	Freelancer	Community-acquired pneumonia	2	60	4
P14	Female	38	Junior high	Sales	Pneumonia	3	120	2
P15	Male	48	Bachelor's	Teacher	ARDS	3	60	4
P16	Male	70	Junior high	Retired	Severe pneumonia	2	60	3
P17	Female	59	Below elementary	Farmer	ARDS	3	60–90	4
P18	Female	74	Below elementary	Farmer	ARDS	3	30–90	6
P19	Male	68	Elementary	Migrant worker	Community-acquired pneumonia	2–3	60–120	4
P20	Female	56	Junior high	Unemployed	Pulmonary infection	4	60	2
P21	Female	76	Elementary	None	AECOPD	3	30–120	3

**Sessions/day**, Daily frequency of prone positioning sessions; **Session/min**, Duration per session in minutes; **Therapy days**, Total duration of prone position therapy in days.

**Figure 1:**
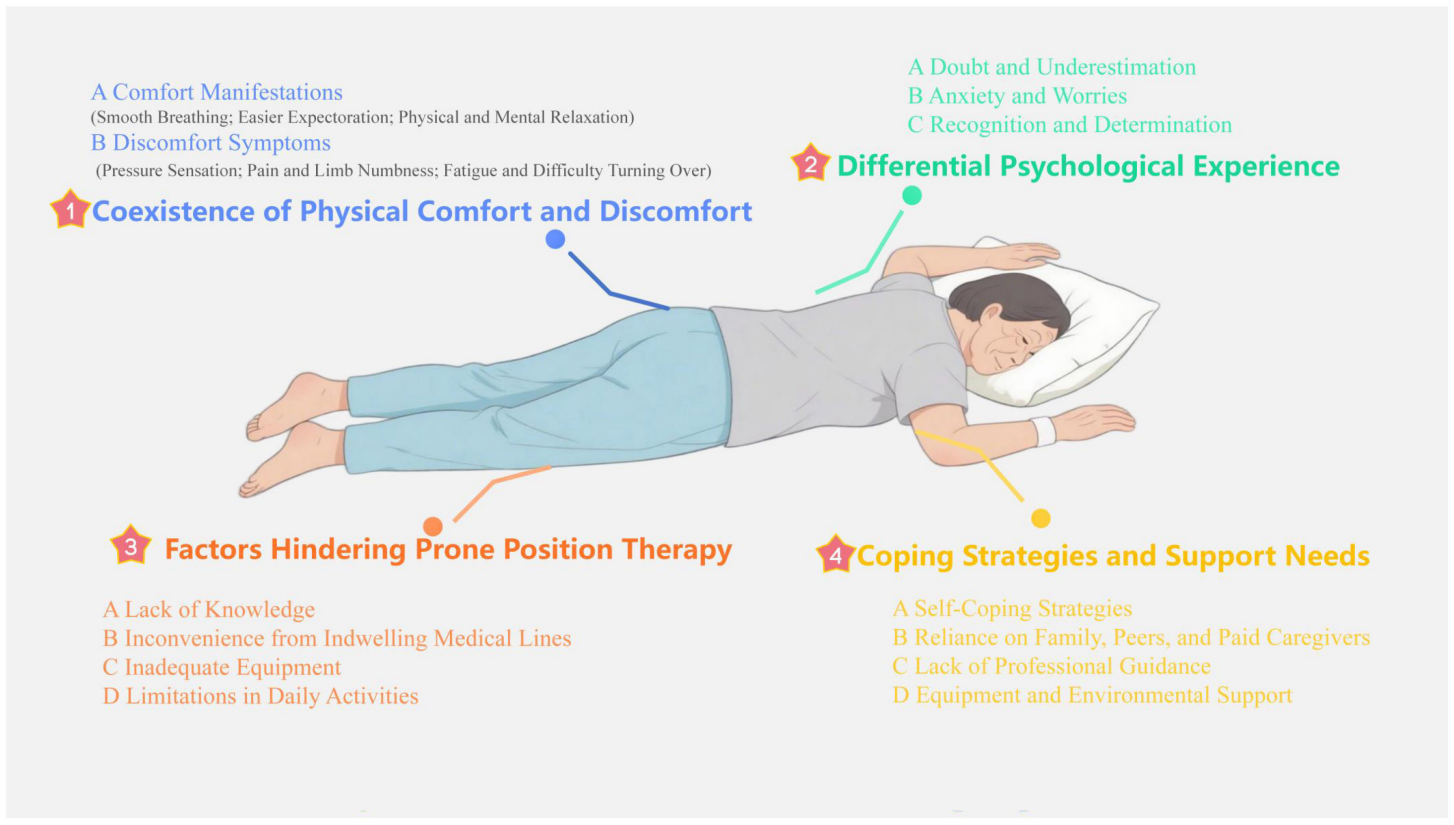
Thematic analysis results of awake prone positioning experiences and needs.

Based on the interview data, the study identified four main themes: (1) Coexistence of Physical Comfort and Discomfort, which refers to the simultaneous experience of both comfort and discomfort during awake prone position therapy; (2) Differential Psychological Experience, emphasizing the varying psychological responses of patients during treatment, including doubt, anxiety, and recognition; (3) Factors Hindering Prone Position Therapy, which involves barriers such as knowledge deficits, the presence of indwelling medical lines, inadequate equipment, and limitations in daily activities that hinder patients from effectively adopting the prone position; (4) Coping Strategies and Support Needs, which explores how patients manage challenges through self-management, family or peer support, professional guidance, and specific equipment needs. The results of these four themes are visually presented in Figure 1.

### 3.1 Coexistence of physical comfort and discomfort

During awake prone positioning, patients experienced a simultaneous coexistence of physical comfort and discomfort. On one hand, they reported improved breathing, easier expectoration, and physical and mental relaxation; on the other hand, they suffered from pressure sensations, pain, limb numbness, fatigue, and difficulty turning over.

#### 3.1.1 Comfortable experiences

Awake prone positioning significantly improves the patient's ventilation status, leading to positive therapeutic experiences, such as smoother breathing, easier expectoration, and overall physical and mental relaxation after the treatment.

##### 3.1.1.1 Smooth breathing

Prone positioning can markedly alleviate dyspnea, reducing discomfort associated with breathing difficulties. Most participants reported smoother breathing after assuming the prone position compared to before. For example, P2 stated, “Before, I felt tired when walking, but after lying prone, I felt less fatigued.” P3 commented, “Breathing is much smoother when I lie prone, but after getting up, I feel fatigued and can barely catch my breath even when walking to the bathroom.” P5 shared, “The effect of lying prone is definitely good. Afterward, I feel more relaxed and breathe better. It is like it helps me breathe.”

##### 3.1.1.2 Easier expectoration

Prone positioning helps clear deep-seated phlegm. Some participants reported that expectoration became easier when lying prone compared to before. P2 noted, “When I was not lying prone; I would cough but couldn't bring up the phlegm. After lying prone, I gradually was able to expectorate more easily.” P6 mentioned, “After lying prone, it became much easier to cough up phlegm, not as effortful as before.” P15 added, “After 2 days, I noticed that phlegm came up more easily, as if my lungs had ‘opened up' a bit.”

##### 3.1.1.3 Physical and mental relaxation

The sense of physical and mental relaxation experienced by some participants enhances their acceptance of awake prone positioning. A few participants reported that after being positioned prone, both their bodies and minds felt more relaxed. P4 shared, “After lying prone and getting up, for the first few minutes, I felt my chest and bones slowly relax, and everything inside seemed more comfortable.” P6 commented, “After lying prone, my body feels much lighter, and I feel more agile than before.” P8 stated, “After lying prone, I feel my body gradually relax after sitting for a while.” P15 said, “I propped my pillow to create a slope, and the airflow seemed to open up the passage. My blood oxygen level increased to 95%, which gave me the confidence to continue lying prone.” P15 also added, “During those few days of prone positioning, I felt my sleep improved slightly.”

#### 3.1.2 Discomfort symptoms

##### 3.1.2.1 Pressure sensation

During awake prone positioning, many participants reported feeling a significant pressure, especially in the chest and abdomen. P5 shared, “The pressure on my chest is uncomfortable. I placed a pillow here (pointing to the chest) and rested my head on my hand. All the weight was focused here, and after lying prone for a long time, I couldn't tolerate it anymore, especially since I weigh over 150 pounds.” P8 remarked, “When lying prone, I felt like something was pressing on my left chest, making it hard to breathe.” P4 noted, “My ribs felt like they were deformed. You can only truly feel it once you experience it yourself.” P13 mentioned, “After lying prone for a while, my stomach feels pressed, and I experience bloating.” P16 added, “I'm 70 years old, and after a long time, it feels even more exhausting.”

##### 3.1.2.2 Pain and limb numbness

The majority of participants reported that prone positioning caused pain in the chest, back, and knees, as well as numbness in the upper limbs. The chest pain was the most prominent. P8 stated, “There's some pain in my chest, as if something is pressing on it. Being older, the bones hurt more after lying prone for a long time.” P5 said, “I can't stand the pressure here (pointing to the chest), but everything else is fine.” P1 shared, “My knees hurt, and my back is sore, and my arms feel numb while lying prone.” P4 mentioned, “If I lie prone for too long, my arms start to feel like they‘ve lost sensation, like they're numb. I can't tolerate it, and I think I won't even be able to last 20 min.”

##### 3.1.2.3 Fatigue and difficulty turning over

Awake prone positioning requires changing positions from supine to prone and vice versa, and some participants reported difficulty turning over and feeling exhausted after large movements. P1 explained, “It's more comfortable lying prone, but after trying to turn over and move, I feel both fatigued and short of breath. If I lie prone incorrectly, I'll be tired for a while, and it can take half a day before I can turn over again.” P9 noted, “As I get older, it's harder to move. At first, I didn't feel tired while lying prone, but after about 20 to 30 min, I started to feel really exhausted.” P10 shared, “When I got up after lying prone, I felt extremely tired. It took more than a minute before I felt better.”

### 3.2 Differential psychological experience

Patients' psychological responses to awake prone positioning varied considerably, ranging from negative emotions to positive acceptance. Initial responses included doubts about effectiveness, lack of attention to the therapy, and anxiety about medical lines; however, after experiencing symptom relief, many patients developed recognition of the treatment's benefits and demonstrated determination to continue.

#### 3.2.1 Doubts and lack of attention

Due to the disease's outcome being related to comprehensive treatment measures, some participants expressed doubts about the effectiveness of prone positioning therapy. Additionally, the discomfort from the disease itself and the long-standing habit of lying supine made it difficult for patients to adapt to the position change, leading to insufficient attention to the therapy. P5 said, “It's hard to say whether lying prone is effective. I‘ve had a lot of fluids infused, but is it the infusion or the prone position that's working? It's hard to tell.” P1 shared, “Initially, I didn't quite believe it. I thought, how can I breathe if I'm already struggling to breathe?” P7 stated, “I'm not used to lying prone; I've always slept on my side or back. Suddenly, lying prone was unbearable. I tried it slowly to see if it would work, but I might not be able to keep it up.” P16 commented, “I'm older, and after lying prone for a long time, my back hurts terribly, especially the next day. Sometimes, I just want it to end sooner.” P17 remarked, “I can't read, so I just nod when the doctor talks to me. I do whatever they ask without questioning whether it's effective.”

#### 3.2.2 Anxiety and worries

During awake prone positioning, the treatment procedure caused patients to experience anxiety and concerns. Most participants expressed that they refrained from lying prone during infusion therapy due to concerns about the IV or oxygen tube becoming disconnected or the needle shifting. P1 said, “When I'm lying prone, I have to be careful about the infusion and oxygen tube getting tangled.” P7 mentioned, “I have severe bronchitis, and I can barely breathe when walking. Lying prone makes me feel a bit nervous.” P8 commented, “The oxygen tube keeps coming out when I lie prone, and I haven't tried lying prone while using oxygen. Infusion also affects me; the needle has fallen out a few times. I always cover the injection site with gauze. I'm getting older, and my diabetes is getting harder to manage, so it's getting more difficult to find veins.” P10 noted, “I didn't lie prone while receiving infusion therapy because the needle was in my clavicle area, and I was afraid of infiltration. Once I turned, the needle fell out.”

#### 3.2.3 Recognition and determination

Recognition of the effects of prone positioning therapy strengthened patients‘ confidence in the treatment. Most participants reported significant improvement in clinical symptoms after undergoing awake prone positioning. P1 shared, “I didn't know it worked this well. I wish I had started earlier. Lying prone on the bed is better than lying on my back. For people like me with lung infections, I will spread the word about this experience.” P2 remarked, “The doctor told me to lie prone, and I wanted to try it. It seemed to help my symptoms a lot. Before, my oxygen saturation was only 91 or 92, but after lying prone, it fluctuates between 98 and 99.” P5 said, “Lying prone for a long time is effective, so I'll keep doing it; it's really beneficial for my breathing.” P10 added, “At first, I could only lie prone for 2 h at most, but now I can manage 3 or 4 h. When I get tired, I rest for a while and then continue. After lying prone, my CT results were much better than when I was in the ICU.” P14 shared, “Prone positioning worked well for me, maybe because I'm younger. After 2 days of lying prone, I felt completely better.”

Psychological responses evolved with clinical experience. P1 shifted from doubt to recognition after symptom relief. P2 gained confidence when SpO_2_ rose from 91%−92% to 98%−99%. P10 extended prone time from 2 to 3–4 h after CT improvement. Conversely, P16 wanted sessions to “end sooner” due to persistent pain without perceived benefit. However, despite these positive responses, several factors hindered patients' ability to maintain prone positioning, as described below.

### 3.3 Factors hindering prone position therapy

Several factors, besides physical discomfort and psychological distress, were found to hinder patients' adherence to awake prone positioning, including a lack of relevant knowledge, inconvenience from indwelling medical lines, inadequate equipment, and limitations in daily activities.

#### 3.3.1 Lack of knowledge

Participants had limited knowledge about prone positioning, including its name, purpose, techniques, and how to position the supporting equipment, and they expressed a strong need for information on these topics. P2 said, “Before coming to the hospital, I had never heard of lying prone. No one at home had mentioned it, and I find it hard to trust health information I see on social media platforms like TikTok.” P13 commented, “I didn't know there were different ways to lie prone other than just lying flat.” P3 noted, “At first, I didn't use any pillow to support my head, and when I woke up, my face was swollen. I had to figure out how to position the pillows by myself.” P4 said, “After being discharged, I'm not sure if I can continue lying prone at home.” P17 shared, “I'm not very educated, so I don't really understand this treatment. At first, I was confused and worried that lying prone might damage my lungs.” P18 said, “A nurse explained it to me, but I only remember that it's good for my lungs. I don't remember the details.”

#### 3.3.2 Inconvenience from indwelling medical lines

Some participants reported that the insertion of medical tubes caused inconvenience during prone positioning therapy. P9 said, “I don't want to lie prone because I have a urinary catheter. It makes it very inconvenient.” P10 remarked, “Lying prone with a mask is uncomfortable. Wearing it is hard enough, and it's even worse when lying prone.” P19 shared, “When lying prone, the tube in my nose (HFNC) always pulls and hurts. Even the slightest movement feels like it's being yanked.” P20 commented, “When lying prone, my biggest worry is not the discomfort, but that the tracheal tube might bend. The airflow fluctuates—sometimes strong, sometimes weak. What if I can't breathe properly?”

#### 3.3.3 Insufficient prone positioning equipment

The lack of proper prone positioning equipment impaired the patients' tolerance to the treatment. Some participants noted that hospitals had insufficient equipment, with each bed only providing one pillow. P4 stated, “The hospital lacks dedicated prone positioning equipment. There's only one pillow, and it can only be placed in one spot. There's nothing else to support the other areas. After a while, it becomes unbearable.” P7 commented, “The hospital only provides one pillow. I used my clothes to prop myself up, but lying prone for too long is uncomfortable.” P18 noted, “I hope the hospital can provide more specialized equipment for lying prone so that it won't be so painful.” P16 added, “I have to roll up my clothes to support my elbows and knees, but it always feels unstable and slides off.”

#### 3.3.4 Limitations on daily activities

Some participants noted that daily activities such as eating, resting, excreting, and entertaining were disrupted, affecting the duration of prone positioning. P12 said, “I find lying prone very inconvenient, especially when drinking or eating. It's not as easy as sitting up to drink or eat; I have to sit up to do those things.” P3 remarked, “Lying prone makes it hard to take naps. You have to be really determined to keep it up. I usually end up lying on my back after a while.” P6 shared, “It's very inconvenient to lie prone when I'm receiving infusion therapy or need to go to the bathroom. I need to change positions frequently, sometimes only being able to lie prone for a short while before getting up to go to the toilet.” P11 commented, “Lying prone makes it difficult to look at my phone. I just can't do it for long without taking a break and getting up.” P16 said, “My back hurts, and I can only manage to stay in the prone position for 20 to 30 min before the pain starts.” P9 remarked, “The light is too bright, and the noise is loud. I find it hard to stay comfortable lying prone for long periods. I'm often woken up during the day when I finally fall asleep. Earplugs and an eye mask would really help.”

### 3.4 Coping strategies and support needs

To manage discomfort and address barriers during awake prone positioning, patients employed various coping strategies and expressed specific support needs. These included self-initiated coping, reliance on family, peers, and paid caregivers, lack of professional guidance, and equipment and environmental support.

#### 3.4.1 Self-coping strategies

Patients employed several coping strategies to manage discomfort during prone positioning, including adjusting their position, taking breaks from prone lying, or pushing themselves until they could no longer tolerate it. P5 shared, “I can only stay in a side-turned position for 20 min, then I quickly turn my head to the other side.” P8 commented, “I just keep switching between my two hands. When I can't stand it anymore, I sit up.” P4 added, “When my hands get numb, I turn to my side and gradually recover. When my ribs hurt, I endure it until it becomes unbearable, then I stop lying prone.” P15 noted, “I try to give myself mental cues, like a teacher, telling myself this is part of my treatment.”

#### 3.4.2 Reliance on family, peers, and paid caregivers

Most participants reported that healthcare professionals were busy and that, during awake prone positioning, small tasks such as adjusting the prone position could be handled by themselves without bothering the medical staff. Family members and fellow patients could assist. P2 said, “I don't need the doctors to help with these small things since they‘re busy. They've told me how to lie prone, so I just do it myself, adjusting the position however I feel comfortable. My family helps cover me with a blanket and alerts the doctor when the infusion is done.” P5 shared, “My children take turns looking after me 24/7. If I'm uncomfortable, they help me turn over or cover me with a blanket.” P1 said, “My roommate helps me turn over, and if something's uncomfortable, they help me adjust.”P16 said, “Since my family members are working, I hired a paid caregiver. They know how to make me more comfortable when I lie prone.”

#### 3.4.3 Lack of professional guidance

Some participants hoped that healthcare workers could provide professional guidance on the steps of prone positioning and on properly arranging supporting equipment. P8 mentioned, “I've been doing it on my own, but it would be helpful if the doctor could show me the proper way to lie down and adjust the pillows.” P9 remarked, “No one taught us how to position the pillows. We just put them wherever they feel comfortable during prone positioning.” P15 shared, “In the regular ward, the doctor told me to lie prone, but the nurses didn't formally guide me. My family watched videos online to learn how to help me.” P21 mentioned, “If someone is around to slowly guide me on how to breathe, I'd be able to stick with it more easily.”

#### 3.4.4 Equipment and environmental support

Participants expressed a need for additional supportive equipment during awake prone positioning. P4 stated, “If I had a small pillow to support my stomach, it would be more comfortable.” P9 remarked, “Although the effects of lying prone are good, I think it would be easier if there were something to support me at the bedside. If I get tired, I could take a break and continue. A machine that helps reposition patients without effort, with a device to support my head, would be great.” P7 shared, “In the beginning, I was in a cramped corridor with a bed added in, and it was difficult to move around. It was much better when I got a room, and a single room would have been even better.” P15 said, “I hope there's specialized equipment to support prone positioning, especially to keep the head and upper limbs supported, and to free up the shoulders and perineum, so that lying prone for long periods won't make my shoulders hurt as much.”

## 4 Discussion

### 4.1 Proper management of discomfort symptoms to promote patient physical comfort

Discomfort during awake prone positioning can hinder its implementation. Our findings show that physical discomfort during awake prone positioning is primarily manifested as chest pressure, pain, and limb numbness, aligning with the findings of Elharrar and Ng et al. (16, 17). While prone positioning can improve oxygenation, reduce the work of breathing, and lower the risk of intubation, sustaining prone positioning remains challenging, with discomfort being a frequent reason for discontinuation (18, 19). Prasad's results also emphasized that comfort acts as a major obstacle to prone treatment (20). Wensley found that comfort in healthcare interactions is closely related to positive doctor-patient relationships, nursing care, and treatment (21). Therefore, healthcare providers should closely monitor discomfort symptoms during prone positioning therapy and intervene proactively. Studies suggest that assisting patients with various position changes, such as reverse Trendelenburg, dolphin position, rodent position, or alternating prone positioning, can both improve patient comfort and tolerance and enhance oxygenation (22–28).

Additionally, factors such as patient age, cognition, body type, comorbidities, comfort, and whether the patient receives encouragement and guidance can affect adherence to awake prone therapy (29–31). Consistent with these findings, older participants in our study (e.g., P9, 88 years; P16, 70 years; P1, 75 years) frequently reported fatigue, difficulty turning over, and prolonged recovery, whereas younger participants (e.g., P14, 38 years) adapted more readily. Optimizing patient tolerance in respiratory interventions is particularly critical for older or physically vulnerable populations (32). Previous studies suggest that shared decision-making models can increase patient involvement and decision satisfaction (33). Therefore, healthcare providers should fully leverage the shared decision-making model during prone positioning therapy, helping patients choose personalized positioning methods tailored to their condition, age, preferences, and needs to enhance comfort (34, 35).

### 4.2 Focus on psychological experience and provide targeted psychological intervention

This study found that participants experienced diverse psychological responses to awake prone positioning, including doubt, anxiety, and eventual recognition. Some struggled to accept the prone position due to discomfort from the disease itself and their long-standing habit of lying supine. However, after experiencing symptom relief, such as alleviated shortness of breath, participants developed a more positive attitude toward awake prone positioning and became more determined to continue. During the COVID-19 pandemic, patients often exhibited anxiety. To address concerns about discomfort from the disease and the inability to tolerate prone positioning, as well as issues such as dislodged intravenous and oxygen tubes during treatment, healthcare providers should dynamically assess the patient's psychological state and intervene promptly. Research has shown that increasing education materials on awake prone positioning can reduce patient anxiety, and early consideration of low-dose anxiolytics can improve tolerance to prone positioning, although respiratory effects should be monitored (36). Additionally, healthcare providers should ensure that all medical lines remain intact and unobstructed when assisting patients with position changes, secure these lines, and conduct regular rounds. Placing the call bell within the patient's reach can help them feel reassured and more comfortable with the treatment (37).

### 4.3 Strengthen communication and guidance to improve patient adherence to prone positioning therapy

Beyond addressing psychological distress, effective communication and practical guidance are essential for improving adherence. The findings of this study indicate that factors such as physical discomfort, psychological distress, disease knowledge, iatrogenic tube placement, treatment activities, and inadequate supporting equipment all negatively affect the success of prone positioning therapy. Participants with lower educational backgrounds, such as P17 (a 59-year-old farmer with below-elementary education) and P18 (a 74-year-old farmer with below-elementary education), often expressed confusion about the purpose of prone positioning. Some participants reported gaining knowledge about awake prone positioning through oral communication from doctors, self-experimentation, or social media like WeChat, but the information was fragmented and incomplete. This finding aligns with the mixed-methods study by Casado Durández et al., which identified knowledge gaps, uncertainty, and the need for guidance as key barriers to adherence. The study also demonstrated how patient engagement evolves through experiential learning in clinical pathways (38). Martínez Lozano et al. demonstrated that integrating evidence-based eHealth education into pulmonary rehabilitation significantly reduced kinesiophobia and improved psychological adherence, suggesting that similar educational strategies may also help address knowledge deficits and anxiety-related doubts about the effectiveness of treatment in awake prone positioning (39). During therapy, improper use of pillows and incorrect positioning contributed to delayed initiation of prone therapy or shorter durations of adherence. Beyond managing discomfort during positioning, the timing of initiation also matters. Previous studies have shown that early initiation of prone positioning (i.e., starting soon after meeting clinical criteria) significantly reduces patient mortality compared to delayed initiation (40–43). Based on prior literature, healthcare providers should guide patients on where to place pillows (e.g., chest, pelvis, knees, and ankles) to reduce pressure on bony prominences (44, 45). Additionally, implementing a prone-position safety checklist and providing guidance before, during, and after the procedure can enhance patient tolerance and adherence (46, 47).

### 4.4 Establish systematic support to enhance patient self-management awareness and self-efficacy

Three sources of support emerged from our data: family, peers, and healthcare providers. This study found that family members and fellow patients play a crucial role in supporting patients during awake prone positioning, consistent with findings from Zhu et al. (30). All participants stated that, given healthcare providers' busy schedules, they would first seek help from family members or fellow patients when experiencing discomfort. This finding directly reflects the actual support needs of patients in the general ward setting. Research has shown that patient tolerance to awake prone therapy is poor, and the strict visitation policies in most hospitals lead to insufficient family support, which increases patient anxiety. The support and encouragement from the healthcare team are essential (36). Slessarev et al. noted that allowing patients to interact with family members during hospitalization encourages more compassionate healthcare (48). Peer support can enhance the development of self-management skills (49, 50). Family support is also vital for improving patients' quality of life, mental health, and disease treatment outcomes (51, 52). This finding is supported by Mena-Marcos et al., who found that caregiver involvement is a key determinant of patient experience in their qualitative study of traumatic brain injury patients and caregivers (53). Based on our data, it may be beneficial for healthcare providers, peers, and family members to collaborate in supporting patients. Encouraging family members to provide emotional support and facilitating patient-to-patient sharing of prone positioning experiences within the same ward may help bolster patients' confidence in overcoming the disease and enhance their self-management awareness and self-efficacy during awake prone therapy.

## 5 Conclusion

The results of this study indicate that awake patients undergoing awake prone positioning exhibit a range of physiological and psychological responses. Patients commonly face issues such as a lack of knowledge, inconvenience caused by iatrogenic tubes, and insufficient prone positioning equipment, and they express a strong need for comprehensive care. To improve patient adherence, healthcare providers should consider patients' individual feelings and needs and implement personalized interventions. Measures such as adjusting positioning and pillow height, providing turning assistance, offering breathing guidance, and ensuring safe companionship can effectively enhance patient comfort, thereby facilitating the therapy process and potentially promoting disease recovery. Therefore, enhancing patient comfort, strengthening support and guidance, and improving adherence may be beneficial for optimizing treatment outcomes. However, this study does not demonstrate causal effects on clinical outcomes such as intubation rates or mortality. While the relationship between patient comfort and disease recovery is plausible, it requires further quantitative investigation.

## 6 Limitations

This study has several limitations. First, the sample was relatively homogeneous, primarily focusing on patients with hypoxemic respiratory failure who required prone positioning, and a population with lower educational and cultural backgrounds. The study was conducted in Guizhou Province, a region in western China with limited healthcare resources and a predominantly low-education population. These cultural and regional factors may influence both patients' understanding of and adherence to prone positioning. Therefore, these results should be applied with caution to populations with differing socioeconomic or cultural contexts. Additionally, the research was conducted at a single hospital, limiting the generalizability of the findings. As the study relied on qualitative interviews, the data was based on patients' subjective experiences, which may introduce bias. Moreover, this study aimed to explore patient experiences and needs, rather than establish causal relationships. Thus, the findings cannot be used to infer causal links between prone positioning therapy and clinical outcomes, such as intubation rates or mortality. Despite reporting the researchers' reflexive positioning in the Methods section, the interviewers' positive clinical experience with prone positioning may have introduced bias during data collection and interpretation. Lastly, future research should include patients with diverse conditions across multiple healthcare settings and integrate quantitative surveys for more comprehensive and diverse data.

## Data Availability

The raw data supporting the conclusions of this article will be made available by the authors, without undue reservation.
